# PD-L1 expression as poor prognostic factor in patients with non-squamous non-small cell lung cancer

**DOI:** 10.18632/oncotarget.17022

**Published:** 2017-04-11

**Authors:** Cuiling Zhou, Jianjun Tang, Huanhuan Sun, Xiaobin Zheng, Zhanyu Li, Tiantian Sun, Jie Li, Shuncong Wang, Xiuling Zhou, Hongliu Sun, Zhibin Cheng, Hongyu Zhang, Haiqing Ma

**Affiliations:** ^1^ Department of Oncology, The Fifth Affiliated Hospital of Sun Yat-sen University, Zhuhai, Guangdong 519000, China; ^2^ Department of Gastroenterology, Cancer Hospital of Jiangxi Province, Nanchang, Jiangxi 330029, China; ^3^ Department of Respiration, The Fifth Affiliated Hospital of Sun Yat-sen University, Zhuhai, Guangdong 519000, China; ^4^ Department of Pathology, The Fifth Affiliated Hospital of Sun Yat-sen University, Zhuhai, Guangdong 519000, China; ^5^ Department of Hematology, The First Affiliated Hospital of Sun Yat-sen University, Guangzhou, Guangdong 510080, China; ^6^ Department of Breast and Thyroid Surgery, The First Affiliated Hospital of Sun Yat-sen University, Guangzhou, Guangdong 510080, China; ^7^ Department of Pathology, University of Michigan, Ann Arbor, MI 48201, USA

**Keywords:** NSCLC, PD-L1, survival, prognostic factor, histologic type

## Abstract

**Objectives:**

The role of programmed cell death ligand 1 (PD-L1) in non-small cell lung cancer (NSCLC), especially according to histologic type, remains controversial. The purpose of this study was to assess PD-L1 expression and its association with overall survival (OS) and clinicopathologic characteristics in NSCLC.

**Materials and methods:**

Formalin-fixed paraffin-embedded specimens were obtained from 108 patients with surgically resected primary NSCLC. PD-L1 expression was assessed via immunohistochemistry using a histochemistry score system. The relationship between OS or clinicopathologic characteristics and PD-L1 expression was evaluated via the Kaplan-Meier method and Cox proportional hazards model, respectively.

**Results:**

Of 108 NSCLC specimens, 44 had high PD-L1 expression, which was highly associated with histologic type (*p* = 0.003). Patients without PD-L1 expression had remarkably longer OS than those with PD-L1 expression (median OS: 96 months vs. 33 months, *p* < 0.001). In the subgroup analysis of non-squamous cell carcinoma, OS was more favorable in those without PD-L1 expression than in those with PD-L1 expression (median OS: 113 months vs. 37 months, *p* < 0.001). Multivariate analysis revealed that PD-L1 expression (95% confidence interval 1.459-4.520, *p* < 0.001), male sex and higher tumor-node-metastasis stage were significantly correlated with shorter OS.

**Conclusions:**

This study demonstrated that PD-L1 expression is an independent prognostic factor for poor survival in NSCLC patients, especially those with non-squamous NSCLC.

## INTRODUCTION

Lung cancer is the leading cause of cancer death worldwide [1]. Although in recent years multidisciplinary therapies have been widely used in patients with non-small cell lung cancer (NSCLC), the overall prognosis of NSCLC remains poor. Previous studies expressed full confidence in the promising prospects of monoclonal antibodies, which can effectively block inhibitory immune checkpoints, among the various immunotherapeutic strategies in lung cancer [2–4]. Preclinical and clinical data showed that monoclonal antibodies can significantly enhance the antitumor immunity of patients [3].

Cytotoxic T-lymphocyte–associated antigen 4 (CTLA-4), a second counter receptor for the B7 family of co-stimulatory molecules, was the first clinically validated checkpoint pathway target. CTLA-4 inhibits T cell activation in initial stages and breaks tolerance of human cancer antigens, many of which are normal self-antigens, yet produces frequent immune-related adverse events [5–7]. More recently, programmed death 1/programmed cell death ligand-1 (PD-1/PD-L1) has become a popular blockade of co-inhibitory immune pathways. PD-1 (also known as CD279), a member of the CD28 family, is expressed on many lymphocytes and negatively regulates their proliferation and activation, such as activated T cells, regulatory T cells, and B-cells [8, 9]. PD-1 has two binding ligands, PD-L1 (B7-H1, CD274) and programmed cell death ligand-2 (PD-L2, B7-DC, CD273), both of which belong to the B7 family [10–12]. PD-L2 is mainly expressed on activated dendritic cells and macrophages. PD-L1 is not only broadly expressed on non-immune cells, such as T cells, B cells, macrophages, and dendritic cells, but it is also upregulated after their activation. Furthermore, researchers have recently discovered the expression of PD-L1 in various tumor cells, including breast cancer, gastric cancer, NSCLC, pancreatic cancer, bladder cancer, cervical cancer, renal cell carcinoma, and melanoma [13–20]. The inhibition of the PD-1/PD-L1 pathway enhances antitumor immunity to prevent tumor cells from escaping from host immune responses, thus providing a promising strategy for specific tumor immunotherapy [21].

Nonetheless, data on the prognostic role of PD-L1 expression in NSCLC tumor cells and its correlation with clinicopathologic characteristics are conflicting. Two previous studies showed that PD-L1 expression was a favorable prognostic factor for overall survival (OS) in NSCLC [22, 23]. However, several meta-analyses indicated that PD-L1 expression was associated with poor OS in NSCLC patients, yet the association of PD-L1 expression with clinicopathologic characteristics, especially histologic type, remained unclear [24–26].

In this study, we used immunohistochemistry (IHC) to assess the prognostic role of PD-L1 in the OS of patients with surgically resected NSCLC and the association between PD-L1 expression and clinicopathologic characteristics, including histologic type, sex, age, and pathologic tumor-node-metastasis (TNM) stage.

## RESULTS

### Patient characteristics

A total of 108 patients with NSCLC were included in the study. The patients’ characteristics are shown in Table 1. All patients underwent surgical resection when first diagnosed with primary NSCLC. The mean follow-up after surgical resection was 48.4 months. The median age of the patients at diagnosis was 55 years (range, 15-75 years), and 59% were male. In our enrolled NSCLC patients, TNM stages I, II, III, and IV at initial diagnosis were identified in 39, 26, 36, and 6 patients, respectively. The tumor grade of one tumor sample was not reported. The tumor histology was squamous cell carcinoma in 34 specimens, adenocarcinoma in 57 specimens, adenosquamous carcinoma in 11 specimens, and other histologic types in 6 specimens.

**Table 1:** Patient characteristics

Characteristic	No. of patients	%
Sex		
Male	64	59.3
Female	44	40.7
Age (in years)		
< 60	63	58.3
≥ 60	45	41.7
TNM stage		
I	39	36.5
II	26	24.3
III	36	33.6
IV	6	5.6
Tumor stage		
pT1	17	16.0
pT2	58	54.7
pT3	27	25.5
pT4	4	3.8
Node metastasis		
pN0	65	63.1
pN1	11	10.7
pN2	27	26.2
Histologic type		
Squamous cell carcinoma	34	31.4
Adenocarcinoma	57	52.8
Adenosquamous carcinoma	11	10.2
Other	6	5.6
Differentiation degree		
Poorly differentiated	20	22.2
Moderately differentiated	62	68.9
Highly differentiated	8	8.9
PD-L1 expression		
Negative	64	59.3
Positive	44	40.7

### Correlations of PD-L1 expression and clinicopathologic characteristics

**Figure 1:**
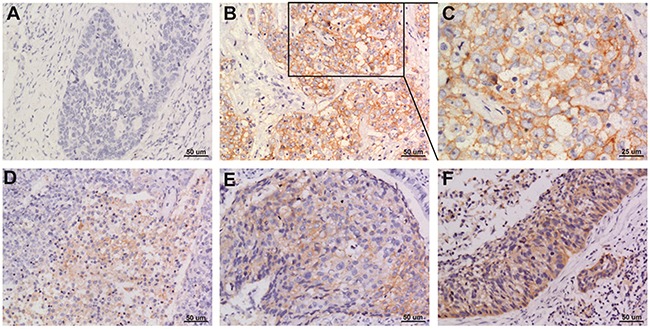
Representative images of PD-L1 staining in squamous cell NSCLC **(A)** negative; **(B and C)** positive; **(D-F)** weak positive, moderate positive, strong positive. Original magnification: A, B, D, E and F panels x 200, C panel x 400.

**Figure 2:**
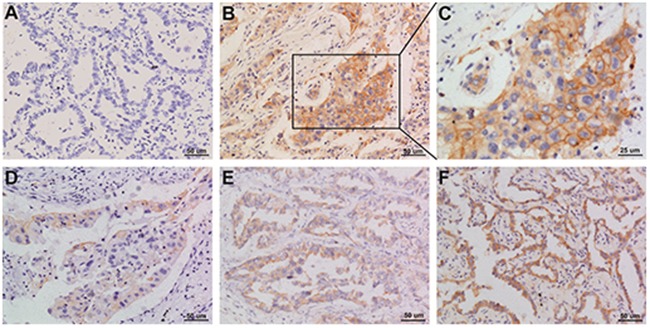
Representative images of PD-L1 staining in adenocarcinoma NSCLC **(A)** negative; **(B and C)** positive; **(D-F)** weak positive, moderate positive, strong positive. Original magnification: A, B, D, E, and F panels x 200, C panel x 400.

Of the 108 cases of NSCLC, 44 had PD-L1 expression in the tumor cell membrane and cytoplasm. Squamous cell carcinomas and adenocarcinomas with PD-L1 staining are shown in Figures 1 and 2.

The correlation of PD-L1 expression with clinicopathologic characteristics is presented in Table 2. PD-L1 expression was significantly associated with histologic type (*p* = 0.003). The percentage of PD-L1-positive cells in squamous cell carcinoma was higher than that in non-squamous cell carcinoma (62.8% vs. 31.1%), whereas, the absence of PD-L1 expression was more common in non-squamous cell carcinoma than in squamous cell carcinoma (68.9% vs. 38.2%). In contrast, PD-L1 expression was not significantly correlated with sex (*p* = 0.408), age (*p* = 0.596), TNM stage (*p* = 0.134), tumor stage (*p* = 0.240), node metastasis (*p* = 0.061), or the degree of differentiation (*p* = 0.065).

**Table 2:** Patients characteristics and their association with PD-L1 expression

Characteristic	Negative, n (%)	Positive, n (%)	*p* Value
Sex			0.408
Male	40 (62.5)	24 (37.5)	
Female	24 (54.5)	20 (45.5)	
Age (in years)			0.596
< 60	36 (57.1)	27 (42.9)	
≥ 60	28 (62.2)	17 (37.8)	
TNM stage			0.134
I/II	42 (64.6)	21 (50.0)	
III/IV	23 (35.4)	21 (50.0)	
Tumor stage			0.240
pT1	13 (76.5)	4 (23.5)	
pT2	33 (56.9)	25 (43.1)	
pT3	16 (59.3)	11 (40.7)	
pT4	1 (25.0)	3 (75.0)	
Node metastasis			0.061
pN0	43 (66.2)	22 (33.8)	
pN1/pN2	18 (47,4)	20 (52.6)	
Histologic type			0.003
Squamous cell carcinoma	13 (38.2)	21 (62.8)	
Non-squamous cell carcinoma	51 (68.9)	23 (31.1)	
Differentiation degree			0.065
Poorly differentiated	8 (40.0)	12 (60.0)	
Moderately differentiated	38 (61.3)	24 (38.7)	
Highly differentiated	7 (87.5)	1 (12.5)	

### 2.3. PD-L1 expression and OS

**Figure 3:**
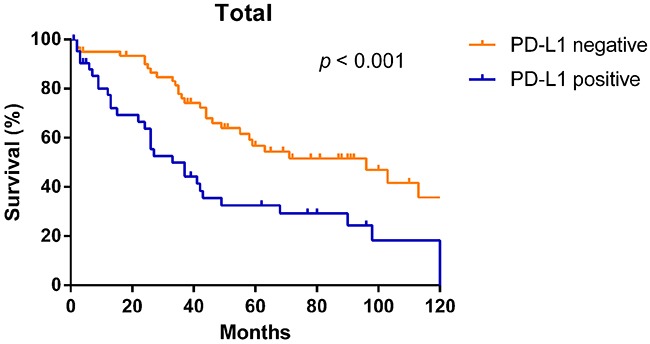
Kaplan–Meier overall survival curves according to PD-L1 expression in NSCLC patients

In Figure 3, the Kaplan–Meier curves revealed that patients whose tumors did not have PD-L1 expression had significantly longer OS than those with PD-L1 expression (median OS: 96 months vs. 33 months, *p* < 0.001).

In univariate analysis, the risk of death was significantly higher in those with PD-L1 expression than in those without PD-L1 expression (95% conference interval (CI), 1.355-3.840, *p* = 0.002). Similar results were observed in those who were male, had a higher TNM stage, had a greater degree of node metastasis, and had squamous cell carcinoma (Table 3). In multivariate analysis, PD-L1 expression (hazerd ratio (HR) = 2.568, 95% CI, 1.459-4.520, *p* < 0.001), male sex (HR = 2.236, 95% CI, 1.236-4.047, *p* = 0.008), and higher TNM stage (HR = 2.416, 95% CI, 1.402-4.164, *p* = 0.001) remained independent risk predictors for poorer OS (Table 3).

**Table 3:** The effect of clinicopathological characteristics and PD-L1 expression on overall survival

Subtype	Hazard ratio (95% CI)	*p* Value
Univariate analysis		
Sex		
Female	1	
Male	2.256 (1.275-3.993)	0.005
Age (in years)		
< 60	1	
≥ 60	1.046 (0.622-1.761)	0.865
TNM Stage		
I/II	1	
III/IV	2.770 (1.635-4.693)	<0.0001
Tumor stage		
pT1	1	
pT2	2.495 (0.971-6.414)	0.058
pT3	3.626 (1.335-9.850)	0.012
Node metastasis		
pN0	1	
pN1/pN2	4.116 (2.308-7.341)	<0.0001
Histologic type		
Non-squamous cell carcinoma	1	
Squamous cell carcinoma	1.754 (1.024-3.004)	0.041
Differentiation degree		
Poorly differentiated	1	
Moderately differentiated	0.969 (0.492-1.908)	0.927
Highly differentiated	0.199 (0.044-0.906)	0.037
PD-L1 expression		
Negative	1	
Positive	2.281 (1.355-3.840)	0.002
**Multivariate analysis**		
Sex		
Female	1	
Male	2.236 (1.236-4.047)	0.008
TNM Stage		
I / II	1	
III / IV	2.416 (1.402-4.164)	0.001
Histologic type		
** Non-squamous cell carcinoma**	1	
Squamous cell carcinoma	1.128 (0.635-2.006)	0.681
PD-L1 expression		
Negative	1	
Positive	2.568 (1.459-4.520)	0.001

**Figure 4:**
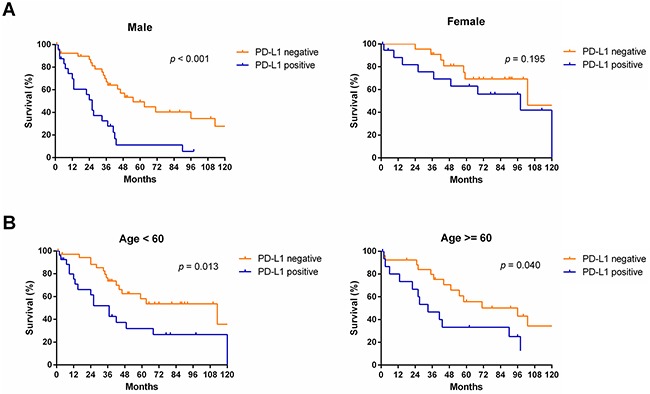
Survival analyses of NSCLC patients with and without PD-L1 expression in subgroups, including, including sex **(A)** and age **(B)**.

**Figure 5:**
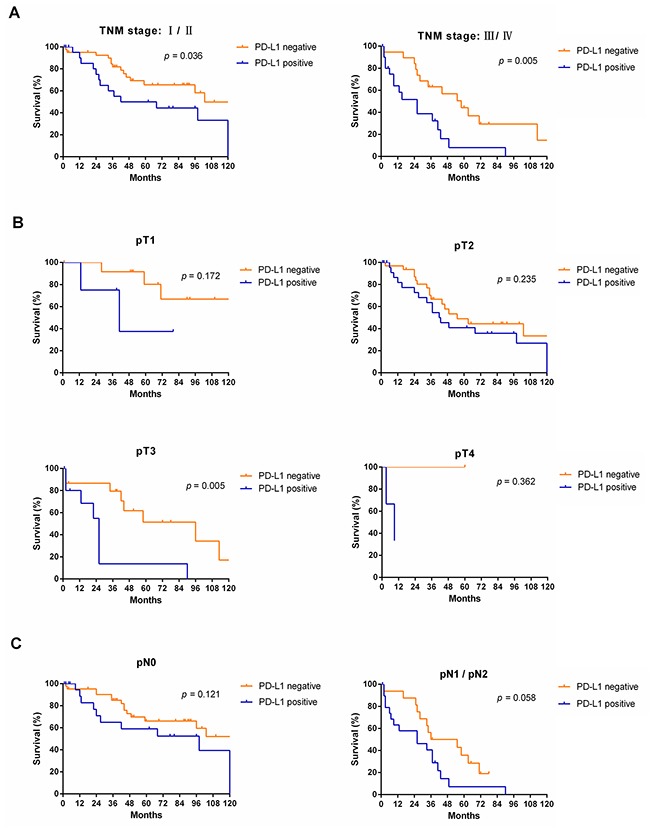
Survival analyses of NSCLC patients with and without PD-L1 expression in subgroups, including TNM stage **(A)**, tumor stage **(B)** and node metastasis **(C)**.

**Figure 6:**
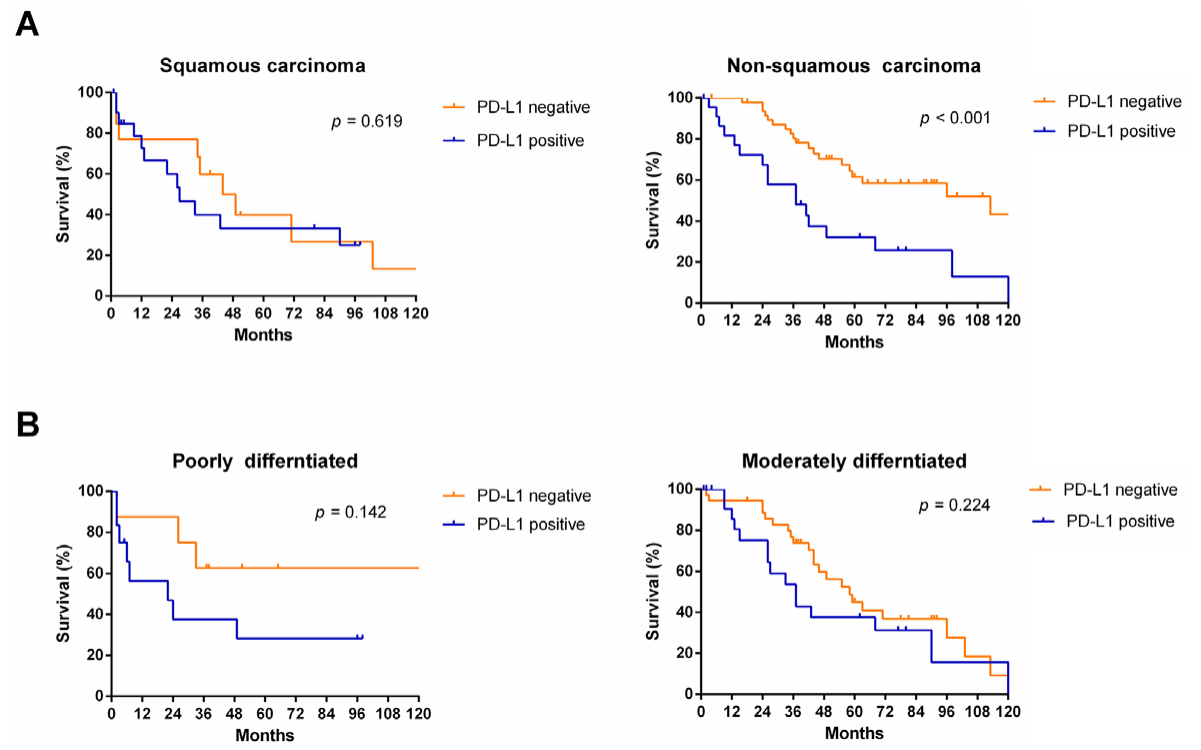
Survival analyses of NSCLC patients with and without PD-L1 expression in subgroups, including histologic type **(A)** and differentiated degree **(B)**.

Subgroup analyses revealed that OS was more favorable in those without PD-L1 expression than in those with PD-L1 expression among males (median OS: 55 months vs. 26 months, *p* < 0.001) and patients of different age groups (< 60, *p* = 0.013; ≥ 60, *p* = 0.040, respectively), but not among females (median OS: 103 months vs. 98 months, *p* = 0.195) (Figure 4). Subgroup analyses also showed that OS was more favorable in those without PD-L1 expression than in those with PD-L1 expression among patients with TNM stage I/II disease (median OS: 103 months vs. 55 months, *p* = 0.036), TNM stage III/IV disease (median OS: 58 months vs. 26 months, *p* = 0.005), and tumor stage pT3 (median OS: 96 months vs. 26 months, *p* = 0.005), but not in patients with tumor stage pT1, pT2, or pT4 or node metastasis (*p* > 0.05) (Figure 5). Interestingly, in the subgroup analysis of non-squamous cell carcinoma, OS was more favorable in those without PD-L1 expression than in those with PD-L1 expression (median OS: 113 months vs. 37 months, *p* < 0.001). However, subgroup analysis of squamous cell carcinoma did not reveal any significant difference in OS between those without PD-L1 expression and those with PD-L1 expression (median OS: 44 months vs. 27 months, *p* = 0.619). Similarly, there was no significant difference based on the degree of differentiation (*p* > 0.05) (Figure 6).

## DISCUSSION

This study demonstrated that PD-L1 expression is an independent prognostic factor for poor OS in NSCLC patients, especially those with non-squamous NSCLC. The analysis also confirmed that high PD-L1 expression is significantly associated with histologic type.

We further demonstrated that patients without PD-L1 expression had significantly longer OS than those with PD-L1 expression. Previous studies similarly reported that high PD-L1 expression was regarded as a poor prognostic biomarker in patients with lung cancer, renal cell carcinoma, breast cancer, malignant melanoma, hepatocellular carcinoma, gastric carcinoma, pancreatic cancer, and ovarian cancer [13, 27–34]. Another study reported that no association was found between PD-L1 overexpression and 3-year overall OS of lung cancer [35]. Using different clone of PD-L1 antibodies as well as different tricks of immunohistochemical technology in the included studies may contribute to the conflicting results. However, further studies are needed to confirm the impact of antibodies on the results of studies.

The PD-1/PD-L1 axis is an inhibitory signaling pathway that gives rise to T-cell exhaustion and inactivation, thus preventing an autoimmune response. PD-L1 expression has also been demonstrated to be related to response to various anti-PD-1/PD-L1 antibodies [36–38]. The phase 3 study CheckMate 057 demonstrated that treatment with nivolumab, the first anti-PD-1 antibody, significantly improved the OS of previously treated patients with advanced non-squamous NSCLC [36]. The supplementary files of CheckMate 057 reported that among the patients with PD-L1 expression levels of 5% or higher, the objective response rate was 36%, and the median OS was 18.1 months. However, among the patients with PD-L1 expression levels of 1% or lower, the objective response rate was only 10%, and the median OS was only 9.7 months. Another randomized controlled trial, Keynote 001, reported that among patients with at least 50% of tumor cells expressing PD-L1 who received the anti-PD-1 monoclonal antibody pembrolizumab, the objective response rate was 45.3%, which means that nearly half of the patients had tumor shrinkage by at least 30%; however, in patients with less than 1% of tumor cells expressing PD-L1, the objective response rate was only 10.7% [37]. Because of the high predictive role of PD-L1 expression, pembrolizumab was approved for patients with PD-L1-positive, advanced NSCLC in 2015. A phase 2 study revealed that PD-L1 expression levels were positive correlated with the efficacy of the anti-PD-L1 monoclonal antibody atezolizumab in NSCLC [38]. For promising clinical benefits may be associated with PD-L1 expression level in early stage treatment, it is pivotal to detecting PD-L1 expression by tumor cell as predictive maker for anti-PD-1/PD-L1 monoclonal antibodies treatment or as prognostic factor for poor survival in NSCLC patients.

In terms of histologic type, similar results were reported by Velcheti *et al*., who found that PD-L1 expression was associated with squamous cell carcinoma [23]. Another study showed that PD-L1 expression was not only associated with adenocarcinoma but also with the degree of differentiation and node metastasis [27]. However, a meta-analysis revealed that none of clinicopathologic characteristics, including sex, smoking status, tumor stage, node metastasis, TNM stage, differentiated degree and histological type was associated with PD-L1 expression in NSCLC [26]. We found no significant difference between smoking status and PD-L1 expression, but considering smoking data in our study was incomplete, so we did not show the smoking data here. A larger sample size with more patients may provide more convincing data.

Furthermore, we found that in patients with non-squamous NSCLC, the OS was longer in those without PD-L1 expression than in those with PD-L1 expression. In contrast, there were no significant differences in different subgroup analyses according to female sex, tumor stage pT1, pT2, or pT4, or node metastasis. Subgroup analyses revealed that OS was more favorable in those without PD-L1 expression than in those with PD-L1 expression among males, but not among females. Cigarette smoking may be one reason of the difference between genders. Smoking is the number one risk factor for lung cancer and it directly contributes to cancer deaths in men more than in women in previous study [39]. The favorable OS in those without PD-L1 patients is observed in TNM stage I/II, III/IV disease and tumor stage pT3, but not in tumor stage pT1, pT2, or pT4 or node metastasis. This may be related to the relatively small sample size in these subgroups in present study. The statistical significance of these differences would be remarkable with a larger sample size. No significant difference in the proportion of squamous carcinoma was observed between the groups with and without PD-L1 expression, although this finding may have been different with longer follow-up or a larger sample size. Our results verified the expression of PD-L1 by tumor cells as a predictive maker in patients with squamous and non-squamous NSCLC. The phase 3 studies CheckMate 017 and CheckMate 057 have reported conflicting results regarding tumor PD-L1 expression and different responses to nivolumab in squamous and non-squamous NSCLC patients. CheckMate 017 reported that OS, response rate, and progression-free survival were not correlative with PD-L1 expression levels in squamous-cell NSCLC patients, but CheckMate 057 reported contrary results in non-squamous NSCLC patients [36, 40].

MEDI4736 is a human IgG1 anti-PD-L1 monoclonal antibody being tested in an ongoing phase III trial in NSCLC. Atezolizumab (MPDL3280A), an engineered IgG anti-PD-L1 antibody, has also shown activity in ongoing phase III trial in NSCLC. Avelumab (MSB0010718C), a fully human anti-PD-L1 IgG1 antibody, currently being investigated in a phase III head-to-head trial of avelumab versus docetaxel in patients with NSCLC [41]. We anticipate promising results from these studies of anti-PD-L1 monoclonal antibodies.

Although NSCLC patients have favorable clinical benefits from blocking the PD-1/PD-L1 pathway, certain issues must still be considered. The most important is that different score systems and different tricks of immunohistochemical technology maybe lead to different results. Furthermore, anti-PD-L1 monoclonal antibodies used for testing PD-L1 expression may lack sensitivity and yield false-negative results, and there is no uniform standard at present. In addition, the expression of PD-L1 is very dynamic, and its uneven distribution in tumor tissue might lead to false-negative results [42–45]. Compared with other detection methods, we evaluated PD-L1 expression in tumor tissues with a standard and credible IHC protocol and reliable histochemistry score (H-score) system, which involved the percentage of positive tumor cells and the intensity of staining in tissue sections.

A standardized method of detecting PD-L1expression maybe provide support for patients who are initially diagnosed with primary NSCLC by allowing for early anti-PD-1 or anti-PD-L1 monoclonal antibody immunotherapy, thus improving treatment efficacy and managing cost. Although the current method of detecting PD-L1 is not as precise as EGFR test, the prospects of PD-L1 as a prognostic factor for poor survival are encouraging.

Efforts were made to conduct sound and rigorous research, but our study had some limitations. First, we obtained several interesting results using samples from 108 patients, but the results will need to be validated with a larger sample size. Second, we retrospectively collected all the information, rather than performing a prospective study. Third, PD-L1 expression was assessed with only IHC, but other detection methods are needed to verify the results.

In conclusion, the current study demonstrated that PD-L1 expression is an independent prognostic factor for poor survival in NSCLC patients, especially those with non-squamous NSCLC. We expect to develop an optimized method of detecting PD-L1 expression in tumor cells to validate PD-L1 as an accurate predictor for anti-PD-1/PD-L1 monoclonal antibody treatment. We anticipate that the anti-tumor efficacy of anti-PD-1/PD-L1 monoclonal antibodies will be strengthened and survival in NSCLC patients will be improved.

## MATERIALS AND METHODS

### Patients and samples

This study included 108 patients who underwent tumor resection or palliative surgery for a primary tumor that was histologically confirmed as primary NSCLC between 2002 and 2012 at the Fifth Affiliated Hospital of Sun Yat-sen University. From their medical records, we retrieved and recorded patients’ clinical data, such as sex, age, histologic type, and TNM stage (using the 7th UICC TNM Staging System of lung cancer). All of the patients were carefully monitored after initial treatment. Expert pathologists at the hospital re-reviewed hematoxylin-eosin–stained slides from all cases, and corresponding formalin-fixed, paraffin-embedded specimens were retrieved, rendering 2-μm-thick pathological sections mounted on glass slides.

### Immunohistochemistry

PD-L1 IHC was performed using the commercial rabbit monoclonal antibody E1L3N (#13684, Cell Signaling Technology, Danvers, USA). Tissue sections were deparaffinized in xylene and a subsequent ethanol series was used to rehydrate them. The sections were unmasked with antigen retrieval buffer (MVS-0099, Maxim EDTA buffer, pH 8.0) in an autoclave for 10 min at 120°C. To block endogenous peroxidase activity, the sections were treated with 0.3% hydrogen peroxide for 30 min. and subsequently washed with phosphate-buffered saline. After being washed with phosphate-buffered saline, the sections were incubated with the PD-L1 antibody at a 1:200 dilution overnight at 4°C. The sections were then washed three times with wash buffer for 5 min each time. The secondary antibody of the EliVision Plus kit detection system and the enhanced polymer 3, 3′diaminobenzidine detection kit (Maxim Biotech, Fuzhou, China) were used according to the manufacturer's instructions. After staining, the sections were washed in distilled water and dehydrated in graded alcohol. Finally, the sections were mounted with coverslips.

### Assessment of PD-L1 expression

All of the stained sections were scored in five randomly selected areas containing tumor cells, which showed membranous and cytoplasmic staining. The percentage of positive tumor cells was graded on a scale of 0-4: 0 (< 1%), 1 (1-10%); 2 (11-50%); 3, (51-70%); and 4 (> 70%). The intensity of staining was scored as follows: 0 (no staining), 1 (weak staining), 2 (moderate staining), and 3 (strong staining). The H-score, ranging from 0 to 12, was calculated by multiplying the percentage of positive tumor cells by the intensity of staining on the tissue sections. The H-scores were categorized as follows: 0: negative (-), 1-4: weak positive (+), 5-8: moderately positive (+ +), 9-12: strong positive (+ + +).

### Statistical analyses

Each clinicopathologic characteristic was evaluated using Pearson's chi-squared test or Fisher's exact test (categorical variables). OS was measured from the date of the initial operation until the date of death or last follow-up. The Kaplan-Meier method and log-rank test were applied to assess OS. Univariate Cox regression proportional hazards analysis was used to assess clinicopathologic characteristics significantly related to OS with HRs and 95% CIs. Multivariate Cox proportional hazards analysis was performed to determine whether PD-L1 expression is an independent prognostic factor. A two-sided *p* value of < 0.05 was considered statistically significant. Several variables were unknown; thus, their implications were not assessed. All statistical analyses were performed using SPSS 20 software (IBM, Armonk, NY).
